# Mobile Health to Maintain Continuity of Patient-Centered Care for Chronic Kidney Disease: Content Analysis of Apps

**DOI:** 10.2196/10173

**Published:** 2018-04-20

**Authors:** Ying-Li Lee, Yan-Yan Cui, Ming-Hsiang Tu, Yu-Chi Chen, Polun Chang

**Affiliations:** ^1^ Institute of Biomedical Informatics National Yang-Ming University Taipei Taiwan; ^2^ Department of Nursing Chi Mei Medical Center Tainan Taiwan; ^3^ Institute of Clinical Nursing School of Nursing National Yang-Ming University Taipei Taiwan

**Keywords:** mobile apps, chronic kidney diseases, self-management, continuity of patient care, patient-centered care

## Abstract

**Background:**

Chronic kidney disease (CKD) is a global health problem with a high economic burden, which is particularly prevalent in Taiwan. Mobile health apps have been widely used to maintain continuity of patient care for various chronic diseases. To slow the progression of CKD, continuity of care is vital for patients’ self-management and cooperation with health care professionals. However, the literature provides a limited understanding of the use of mobile health apps to maintain continuity of patient-centered care for CKD.

**Objective:**

This study identified apps related to the continuity of patient-centered care for CKD on the App Store, Google Play, and 360 Mobile Assistant, and explored the information and frequency of changes in these apps available to the public on different platforms. App functionalities, like patient self-management and patient management support for health care professionals, were also examined.

**Methods:**

We used the CKD-related keywords “kidney,” “renal,” “nephro,” “chronic kidney disease,” “CKD,” and “kidney disease” in traditional Chinese, simplified Chinese, and English to search 3 app platforms: App Store, Google Play, and 360 Mobile Assistant. A total of 2 reviewers reached consensus on coding guidelines and coded the contents and functionalities of the apps through content analysis. After coding, Microsoft Office Excel 2016 was used to calculate Cohen kappa coefficients and analyze the contents and functionalities of the apps.

**Results:**

A total of 177 apps related to patient-centered care for CKD in any language were included. On the basis of their functionality and content, 67 apps were recommended for patients. Among them, the most common functionalities were CKD information and CKD self-management (38/67, 57%), e-consultation (17/67, 25%), CKD nutrition education (16/67, 24%), and estimated glomerular filtration rate (eGFR) calculators (13/67, 19%). In addition, 67 apps were recommended for health care professionals. The most common functionalities of these apps were comprehensive clinical calculators (including eGFR; 30/67; 45%), CKD medical professional information (16/67, 24%), stand-alone eGFR calculators (14/67, 21%), and CKD clinical decision support (14/67, 21%). A total of 43 apps with single- or multiple-indicator calculators were found to be suitable for health care professionals and patients. The aspects of patient care apps intended to support self-management of CKD patients were encouraging patients to actively participate in health care (92/110, 83.6%), recognizing and effectively responding to symptoms (56/110, 50.9%), and disease-specific knowledge (53/110, 48.2%). Only 13 apps contained consulting management functions, patient management functions or teleconsultation functions designed to support health care professionals in CKD patient management.

**Conclusions:**

This study revealed that the continuity of patient-centered care for CKD provided by mobile health apps is inadequate for both CKD self-management by patients and patient care support for health care professionals. More comprehensive solutions are required to enhance the continuity of patient-centered care for CKD.

## Introduction

### Continuity of Patient Care in Chronic Kidney Disease

Chronic kidney disease (CKD) is a global health problem with a high economic burden [1,2]. CKD is often accompanied by chronic vascular disease, diabetes, and other comorbidities associated with long-term conditions. Therefore, it is vital for patients with CKD to have continuity of care [3]. The prevalence of CKD globally is approximately 13.4% (11.7%-15.1%). However, the prevalence rate of CKD stages 3-5 is 10.6% (9.2%-12.2%) [4]. According to the latest US Renal Data System annual data report, the incidence and prevalence of end-stage renal disease in Taiwan was the highest in the world in 2015 [5]. Therefore, aggressive and effective interventions are especially crucial in Taiwan for managing patients with CKD.

Since 2001, the US Institute of Medicine has made “patient-centered” 1 of the 6 targets for improving health care [6]. CKD patient-centered medical services aim to improve the collaboration among the health care professionals (HCPs), patients, and primary caregivers to align patient care with patient values and preferences through shared decision-making. Such services focus on HCPs’, patients’, and primary caregivers’ perspectives [7-9]. Depending on the disease characteristics, care methods, and settings, the importance of each type of continuity also differs [10]. Improving continuity is an avenue worthy of exploration to improve patient-centered care for CKD.

### Mobile Apps for Chronic Disease Self-Management

Many systematic reviews have examined the effects of self-management strategies in care for chronic diseases, such as cystic fibrosis [11], osteoarthritis [12], chronic obstructive pulmonary disease [13,14], asthma [15], and chronic pain [16]. However, these studies have agreed that evidence supporting the self-management of chronic disease care as an effective strategy is insufficient. More rigorous studies are required for confirmation. Nevertheless, self-management strategies remain one of the most frequently recommended programs for CKD care [17,18]. Studies have found that CKD self-management education gradually incorporates a patient-centered and individualized management plan [8,19-21]. However, the effectiveness of these individual management plans for CKD care remains to be observed. Some studies have explored the views of all levels of HCPs on CKD patient management and found that primary HCPs feel more challenged when managing complex comorbidities or older patients with CKD. Therefore, enhancing the coordination and continuity of care and using a multidisciplinary team are necessary for improvements in care quality [22,23].

Mobile health apps are widely used for illness prevention, healthy lifestyles, finding HCPs or facilities, diagnosis, education, filling prescriptions, compliance, diabetes care, mental health and behavior disorders care, musculoskeletal system and connective tissue care, oncology care, nervous system care, women’s health care, and children’s health care [24]. A literature review of interactive telemedicine care has indicated that no difference exists between interactive telemedicine care and face-to-face or telephone interviewing in the management of heart failure, but the interactive telemedicine can improve the control of glycemic control for diabetics. However, due to limited data, the costs of an interactive telemedicine service and its acceptability to patients and HCPs remain unclear. The authors concluded that the outcomes of such studies depend on the participants, their disease histories, the severity of the diseases, and the chosen interventions [25]. This review indicated that, in addition to the application of technologies, patient-centered and individualized interventions may be critical factors influencing the effectiveness of mobile health or telemedicine.

### Mobile Information Technology for Chronic Kidney Disease

Meeting the care standards set by Kidney Disease Improving Global Outcomes is challenging under the often limited manpower available for CKD primary care. Consequently, applying information technology is vital to optimizing the management of CKD patients in primary care [26-28]. Some studies have used telemedicine in CKD care for patient education, interactive counseling for HCPs, monitoring the effectiveness of patient blood pressure control, and a small proportion of home-based case management [29-32]. Diamantidis and Becker reviewed the use of mobile technology in CKD patient care and revealed that, from early mobile messaging and internet connections to current mobile apps, accessibility of devices, usability of apps, and novelty were the primary factors affecting users’ willingness to continue using such technology. They observed that most CKD patients expected to increase their contact with physicians through an interactive system. Therefore, providing a HCP platform such as Happitque for patient education or patient management is becoming a requirement [33]. Developing an interactive telemedicine system that allows patients to communicate with health care providers is becoming a popular form of intervention through which personal health records, laboratory results, and appointment schedules can be obtained. Even wearable devices and apps that can measure renal function are under development [28]. CKD patient management with a mobile health app is thus a groundbreaking patient-centered approach to designing individualized management plans.

Havas et al explored what support CKD patients required for self-management. They summarized the following 10 aspects of CKD self-management: disease-specific knowledge, managing medications, engaging and sustaining social support, maintaining social and occupational roles, modifying lifestyle, developing and sustaining a positive attitude and caring for mental and physical well-being, building and sustaining effective relationships with health care providers, establishing routine and planning ahead, actively participating in health care, and recognizing and effectively responding to symptoms [8,21]. Many studies have explored the content and functionalities of apps for various diseases or health management [34-39]. However, no studies have specifically examined the functionalities of mobile health apps downloaded from popular mobile app platforms for the continuity of patient-centered care for CKD.

Thousands of health apps are available on popular mobile app platforms (eg, the App Store and Google Play). These platforms are the primary source for many patients or HCPs looking for self-management or disease management apps. In recent years, China’s app market has rapidly developed [40], with 360 Mobile Assistant (25%), Tencent (25%), Baidu (17%), and Xiaomi (13%) occupying approximately 70% the market share among China’s top 10 Android app stores [41]. Among these, 360 Mobile Assistant has gained attention for its security apps. However, the reference or validity descriptions of these apps are rarely displayed on these popular mobile app platforms. Therefore, users are unable to determine whether apps have reliable references and whether they are based on effective health management theories (eg, behavior change techniques). As a result, more studies have explored the gap between the functionalities of apps and evidence-based practice [36,42,43].

Many apps are short-lived; Larsen et al have conducted a longitudinal study of mental health apps and found that the environment for such apps is unstable. More than half of the studied apps were revised roughly every 4 months, and on average, 1 app was deleted for every 2.9 days [44]. Zaidan and Roehrer investigated diet and weight loss apps and discovered that some app developers on Google Play release apps with different names but identical content. They also noted that the search algorithm used by Google Play focused on titles and keywords of apps rather than content; however, titles and keywords may not always reflect the content of the apps [39]. This behavior may increase users’ difficulty in selecting apps.

### Objective

This study aimed to: (1) identify the current apps related to the continuity of patient-centered care for CKD on the App Store, Google Play, and 360 Mobile Assistant and explore the information and frequency of changes in these apps available to the public on different platforms; (2) analyze the functionalities and recommended users of apps related to the continuity of patient-centered care for CKD; (3) compare the functionalities provided by apps related to the continuity of patient-centered care for CKD according to 10 aspects of requiring additional support in patient self-management for CKD and the functionalities supporting HCPs for implementing CKD patient management.

## Methods

### Identifying Apps Related to Patient-Centered Care for Chronic Kidney Disease

A total of 3 reviewers (YLL, YYC, and MHT) conducted this study. All reviewers are experts in nursing and mobile health care. Moreover, 2 were responsible for searching for apps as well as coding the features and functionalities. The third senior expert was responsible for monitoring and moderating the results of the data collection. From March to April 2016, 2 reviewers (YLL and YYC) searched 3 app platforms, namely, the App Store, Google Play, and 360 Mobile Assistant, using the CKD-related keywords “kidney,” “renal,” “nephro,” “chronic kidney disease,” “CKD,” and “kidney disease” in traditional Chinese, simplified Chinese, and English. This study only included apps related to patient-centered care for CKD that could be used on mobile phones or tablets; those designed for journals, medical conference manuals, medical practice guidelines or reference materials, clinical comprehensive computers, and hemodialysis were excluded, as were those that could be not be installed or operated.

### Developing Coding Guidelines

This study was conducted through content analysis. First, 1 reviewer (YLL) drafted coding guidelines according to the outlined aims and previous studies assessing health apps [38,39,45]. Following a review, iterative assessing, revising, and testing were conducted for 10 apps randomly selected from those identified in the first stage, and coding guidelines were confirmed (including the definition of each evaluation element and a coding example). The coding guide divided the evaluation elements into the following categories: (1) information provided by the developer, namely, app platform, language, price, content rating (Google Play and App Store only), installation times (Google Play and 360 Mobile Assistant only), privacy policy, registration requirements, last release date, current version, Android or iOS minimum version requirements, description for certification of medical grade app (such as the US Food and Drug Administration (FDA) [46] or MEDDEV 2.1/6 guidelines for medical devices in the European Union [47]); (2) users’ rating, namely, current rating (Google Play and 360 Mobile Assistant only) and the total number of ratings (Google Play only); and (3) researchers evaluation, namely, the classification of the contents and functionalities, recommended users (HCPs, patients, or both), description of reference, and providers (eg, medical profession–related organizations).

### Coding the Features and Functionalities of Apps

Before formal coding, all reviewers obtained coding consensus according to the coding guidelines. First, 2 reviewers (YLL and YYC) identified the top 30 apps from the first stage, 23 for Google Play, 15 for 360 Mobile Assistant, 13 for the App Store, and 1 not included in the final sample. All information provided on the app platforms by their developers was documented. They then downloaded, installed, and operated the apps before coding independently according to the coding guidelines.

**Table 1:** Interrater reliability for all variables.

Variable	Cohen kappa coefficient
App platform	.67
Language	.77
Price	.83
Privacy policy	.71
Registration requirements	1
The classifications of the contents and functionalities	.65
Recommended users	.86
Description of reference	.81
Developers	.81
Description for certification of medical app	1

The apps that could not be registered and authorized were coded according to the screenshots and descriptions of features on the app platforms. The coding results were then recorded in an Excel worksheet.

After coding, the Cohen kappa coefficient of each variable was calculated using Microsoft Office Excel 2016 to test the interrater reliability (Table 1). The Cohen kappa coefficients ranged from .65 (the classifications of the contents and functionalities) to 1 (registration requirements and description for certification of medical app), and the levels of agreements were from moderate to strong [48]. For the differences, all reviewers discussed until consensus was reached and revised the latest coding guidelines accordingly.

After obtaining consensus, 2 reviewers (YLL and YYC) examined the contents and functionalities of all apps individually and encoded them according to the latest coding guidelines. After coding, the 2 reviewers (YLL and YYC) agreed on the coding results through comparison and discussion. If consensus could not be reached, the third senior expert (MHT) participated in the discussion to confirm the final coding.

The baseline survey was conducted between April and June 2016. To reveal the follow-up maintenance status and loss rate of apps included in the baseline survey, 1 reviewer (YLL) conducted a second survey in December 2016, a third survey in March 2017, and a fourth survey in June 2017. The follow-up surveys recorded whether the app was downloadable, the number of app downloads, the date of the last release, and the user rating from each app platform.

### Comparing Functionalities of Apps According to 10 Aspects of Chronic Kidney Disease Self-Management

To compare the functionalities of patient-centered care apps for CKD according to the 10 aspects of CKD self-management, 1 reviewer (YLL) categorized the 110 apps suitable for patients according to the 10 support aspects introduced by Havas et al; the categories were discussed with the other researchers to reach a consensus [8,21]. Each functionality category corresponded to more than 2 aspects. The quality of the information from the mobile apps was analyzed with reference to previous studies [36,49]. Each aspect was regarded as a quality indicator. When the functionalities of an app contained one of the aspects, a score of 1 was allotted; otherwise, a score of 0 was given. The highest possible score was 10.

### Statistical Analysis

Microsoft Office Excel 2016 was used to conduct descriptive statistical analyses of the app classification properties.

## Results

### App Search Results

**Figure 1:**
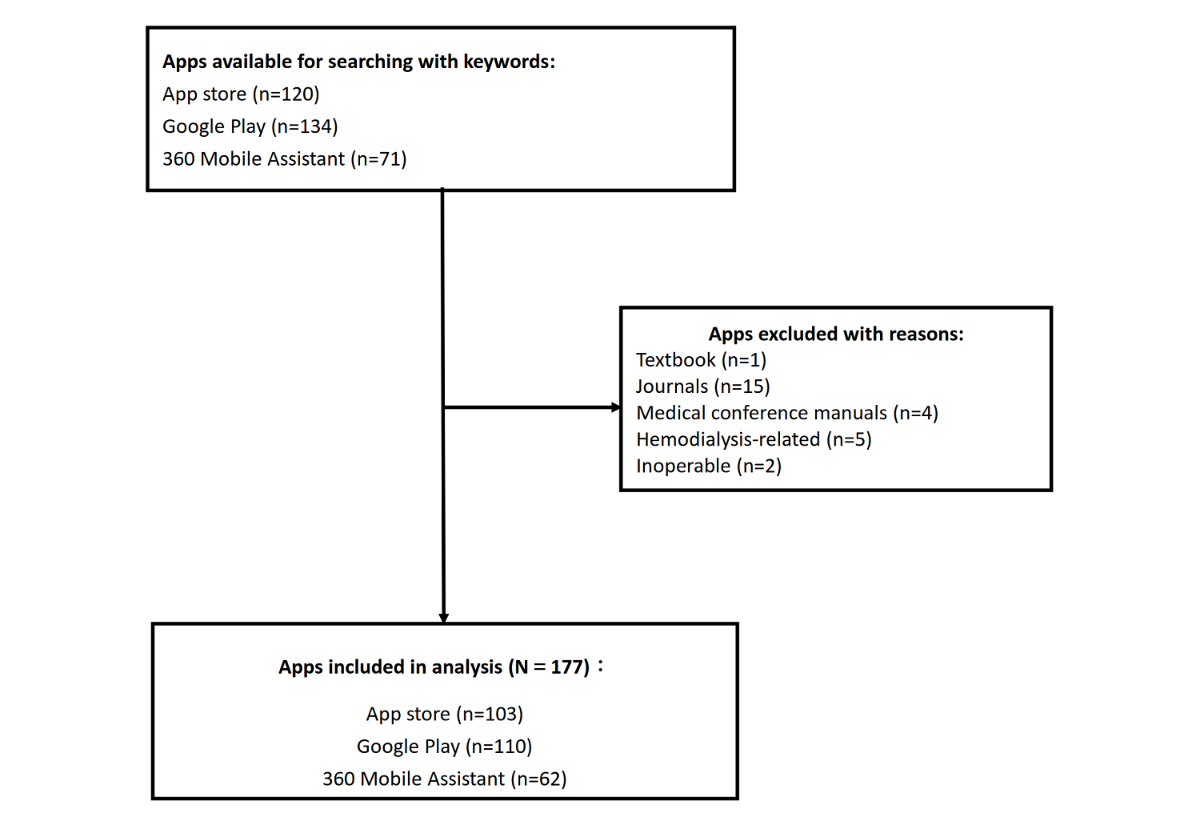
Apps exclusion chart.

A total of 120 apps from the App Store, 134 apps from Google Play, and 71 apps from 360 Mobile Assistant were located. After removing duplicate apps, 204 apps remained. The default exclusion criteria were applied, after which 177 patient-centered care apps for CKD with any language were retained for analysis (Figure 1). Some apps were released on more than 1 platform simultaneously. Among these apps, 58.2% (103/177) were released on the App Store, 62.2% (110/177) on Google Play, and 35.0% (62/177) on 360 Mobile Assistant. The most common language was English (92/177, 52.0%), followed by simplified Chinese (33/177, 18.6%) and multiple languages (16/177, 9.0%). The majority of the apps (123/177, 69.5%) were free and 40.7% (72/177) required registration. A total of 148 apps were developed by non-HCP organizations. In addition, 80.2% (142/177) apps had content that referenced medical literature, and most apps included estimated glomerular filtration rate (eGFR), creatinine clearance rate (CCR), and other calculation formulas; 35 apps referenced no literature. These apps mostly provided CKD information, CKD nutrition education, self-management advice, and other functionalities. None of them were developed by HCP organizations.

Different app platforms provide users with different information regarding hosted apps. The relevant analysis is presented in Multimedia Appendix 1. On the App Store, 62 apps were free (62/103, 60.2%), and the content ratings of 47 apps were “4+.” Most apps did not have user ratings, and no installation times and privacy policies were declared on this platform. Most apps were free on Google Play (81/110, 73.6%). The content ratings for 38 apps were “high maturity” (38/110, 34.6%) and the remaining apps were rated as “3+.”

Nearly half of the apps were not rated and only 4 of the apps were rated with scores of less than 3. Only 14 apps had more than 10,000 downloads, and 19 had privacy policies. All apps on 360 Mobile Assistant were free to download. A total of 35% of the apps were in simplified Chinese. The percentage of simplified Chinese apps on 360 Mobile Assistant was higher than those of App Store (25/103, 24.3%) and Google Play (1/110, 0.9%). Total downloads for most apps did not exceed 10,000. Most apps had no current rating, content rating, or privacy policy. All apps examined in this study had no descriptions of medical grade app certification issued by any government agency. A total of 7 pairs of apps suites (14 apps) had patient and HCP interoperability.

### App Content Analysis

We coded each app based on its content and functionalities and analyzed its recommended users and functionalities (Table 2). Of 177 apps, 67 apps were suitable for patients (patient apps). The most common functionalities of the patient apps were CKD information, CKD self-management, e-counseling, CKD nutrition education, and eGFR calculation. A total of 67 apps were more suitable for HCPs (HCP apps). The most common functionalities of the HCP apps were a comprehensive clinical calculation (including eGFR), provision of CKD medical professional information, stand-alone eGFR calculation, and clinical decision support for CKD. However, the designs of the decision support functionalities were mostly basic. Among the HCP apps, 7 apps contained management consultation functionality, 5 contained patient management functionality, and only 1 contained teleconsultation functionality. A further 43 apps had main functionalities including simple eGFR calculation, CCR calculation, and CKD staging, which were suitable for both HCPs and patients.

**Figure 2:**
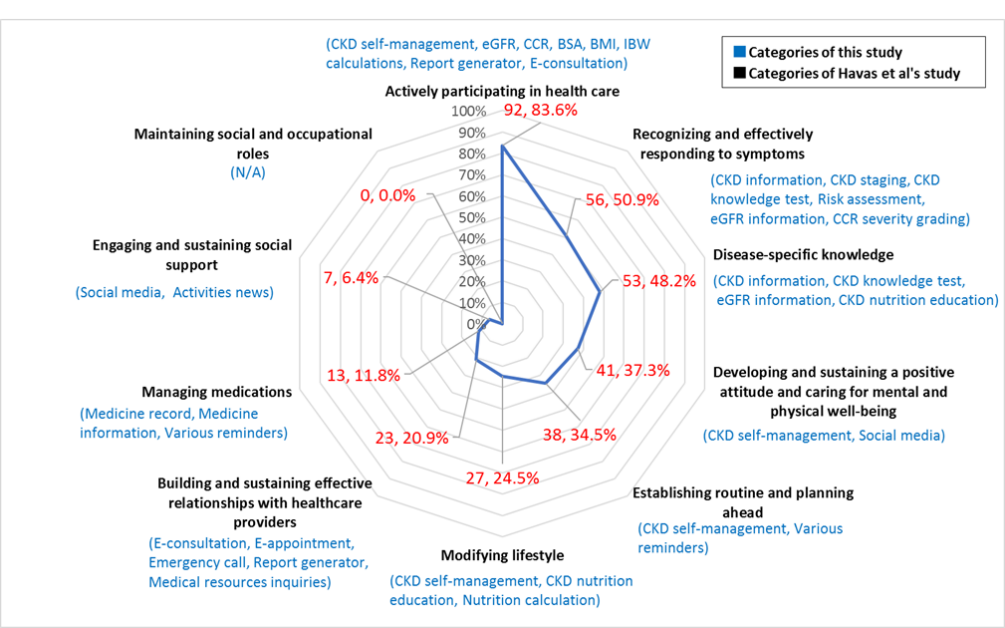
Patient apps categories based on the 10 support aspects of chronic kidney disease (CKD) patient self-management (N=110). BMI: body mass index; BSA: body surface area; CCR: creatinine clearance rate; eGFR: estimated glomerular filtration rate; IBW: ideal body weight; N/A: not applicable.

We categorized and scored 110 patient apps based on the 10 support aspects of patient self-management for CKD (Figure 2) [21]. The highest score received was 8 (the app met 8 aspects of CKD self-management), and the lowest score was 1. Among these apps, 62 received scores ranging from 1 to 3 (56.4%), 39 apps ranged from 4 to 6 (35.5%), 8 apps scored 7 (7.3%), and only 1 app received a score of 8 (2.0%). The most common functionalities were supporting patients actively participating in health care (92/110, 83.6%), recognizing and effectively responding to symptoms (56/110, 50.9%), and disease-specific knowledge (53/110, 48.2%). Other apps were designed for managing medications (13/110, 11.8%) and engaging and sustaining social support (7/110, 6.4%). None of the apps met the maintaining social and occupational roles aspects. The detailed features of each app are listed in Multimedia Appendix 2.

We determined that 38 patient apps provided continual recording of various data for self-management purposes. Only 7 pairs were simplified Chinese app suites (14 apps) developed by Chinese developers for both patients and HCPs that provided patient and HCP interoperability. One of the apps could be linked with wearable devices for instant monitoring of the patient’s important health information such as heart rate and blood pressure for transmission back to the physician’s app.

**Table 2:** Overview of the recommended users and functionalities of apps (N=177). CCR: creatinine clearance rate; CKD: chronic kidney disease; eGFR: estimated glomerular filtration rate; HCP: health care professionals.

Functionalities	Patients (N=67), n (%)	HCPs (N=67), n (%)	Both (N=43), n (%)
CKD information	38 (57)	3 (4)	1 (2)
CKD self-management	38 (57)	0 (0)	0 (0)
E-consultation	17 (25)	0 (0)	0 (0)
CKD nutrition education	16 (24)	0 (0)	1 (2)
eGFR calculation	13 (19)	14 (21)	33 (77)
E-appointment	8 (12)	0 (0)	0 (0)
Various reminders	7 (10)	0 (0)	0 (0)
Social media	6 (9)	1 (1)	0 (0)
CKD staging	5 (7)	6 (9)	10 (23)
Medicine information	3 (4)	0 (0)	0 (0)
Nutrition calculation	2 (3)	0 (0)	2 (5)
Medical resources inquiries	2 (3)	0 (0)	0 (0)
CCR calculation	1 (1)	1 (1)	11 (26)
CKD knowledge test	1 (1)	0 (0)	0 (0)
Report generator	1 (1)	0 (0)	1 (2)
Activities news	1 (1)	0 (0)	0 (0)
Risk assessment	1 (1)	3 (4)	0 (0)
Emergency call	1 (1)	0 (0)	0 (0)
Privacy management	1 (1)	0 (0)	0 (0)
Comprehensive clinical calculation (including eGFR)	0 (0)	30 (45)	0 (0)
Provision of CKD medical professional information	0 (0)	16 (24)	0 (0)
Clinical decision support for CKD	0 (0)	14 (21)	0 (0)
Consultation management	0 (0)	7 (10)	0 (0)
Patient management	0 (0)	5 (7)	0 (0)
Body surface area calculation	0 (0)	3 (4)	4 (9)
Body mass index calculation	0 (0)	2 (3)	3 (7)
Other clinical decision support	0 (0)	2 (3)	0 (0)
Appointment reminder	0 (0)	2 (3)	0 (0)
CKD evaluation	0 (0)	1 (1)	0 (0)
Provision of CKD nutritional care professional information	0 (0)	1 (1)	0 (0)
eGFR information	0 (0)	1 (1)	4 (9)
Ideal body weight calculation	0 (0)	1 (1)	4 (9)
Appointment management	0 (0)	1 (1)	0 (0)
Teleconsultation	0 (0)	1 (1)	0 (0)
CCR severity grading	0 (0)	0 (0)	1 (2)

According to the Mobile Medical Applications Guidance of the FDA [46] and the MEDDEV 2.1/6 guideline [47], this app could be governed by the FDA regulations for mobile medical apps or the regulations of the Medical Device Directive 93/42/EEC. However, the developer made no mention in the app of whether the app requires relevant certification.

### Longitudinal Analysis

After completing the baseline survey in June 2016, we conducted 3 follow-up surveys of the 177 apps to understand the loss and update frequency of such apps. We also simultaneously searched for new apps with the same keywords and analyzed the results. In December 2016, 170 apps were determined with 9 removed and 2 new released. In March 2017, 161 apps were determined with 11 removed and 2 new released. In June 2017, we reviewed the status of 165 apps updates on each platform since March 2017 and determined that 4 apps had been removed, 8 apps had been released, and 11.8% (19/161) of apps had been updated, of which 17% (14/84) of apps had been updated on the App Store and 10% (5/50) on 360 Mobile Assistant. The lowest update rate was 8.93% (10/112) on Google Play.

## Discussion

### Principal Findings

In this study, we investigated 3 mobile app platforms, the App Store, Google Play, and 360 Mobile Assistant, and found 177 patient-centered care mobile apps for CKD. Most of the apps were free and did not require registration, which can increase the willingness of users to download the apps and helps to promote usage of patient-centered care apps for CKD. Among the platforms, Google Play provided the most categories of app information. The App Store did not provide total downloads. No content rating and little current rating information were provided on 360 Mobile Assistant. Although most apps did not require registration, many apps did, and they asked patients to provide self-management information without an adequate user privacy protection policy. Studies have demonstrated that many mobile medical apps are insufficiently safe [50,51]. Therefore, providing an adequate privacy protection policy and app security measures is critical for safeguarding users. However, neither the App Store nor 360 Mobile Assistant provided privacy announcements, and although Google Play provided this information, only 19 apps included privacy policies. Overall, the integrity of the information provided by patients to these patient-centered care apps was inadequate on each platform.

The App Store and Google Play provided content ratings, but we found that content ratings for apps with the same content on these 2 platforms were inconsistent. Some apps are released on more than 1 platform, which can increase app accessibility; however, app versions differ between platforms, providing inconsistent experiences. Zaidan and Roehrer noted that some app developers release apps on Google Play under different names but with the same content. Consequently, when users search for an app using a specific keyword, the returned apps may not meet their requirements [39]. These phenomena were revealed in this study. The gathered data indicated that app developers must enhance management of version updates, content ratings, and app naming consistency between different platforms.

Google Play was the only platform on which 14 apps were installed over 10,000 times. These apps were all related to clinical calculators (eg, eGFR, CCR, and BMI) and contained numerous medical terminologies that are usually suitable for HCPs. With training, patients can also use these apps for self-management; however, apps on all platforms usually fail to tailor themselves to the average user. According to our study, the app functionalities that apply to patients and HCPs are different. Among the 10 support aspects required for self-management of CKD [8,21], current CKD-related apps lacked not only functionalities in “modifying lifestyle,” “building and sustaining effective relationships with health care providers,” “managing medications,” and “engaging and sustaining social support” but also any functionality that met the “maintaining social and occupational roles” aspect. Maintaining social and occupational roles includes continuing to work, sustaining hobbies, maintaining relationships, and home roles. Maintaining social and occupational roles has a marked impact on health and well-being [52]. Under the pressure of a long-term battle with CKD, middle-aged patients require more support to maintain social and occupational roles. For the future development of patient self-management apps for CKD, we recommend strengthening the functionality of these aspects to meet patients’ needs and achieve patient-centered CKD care. Some studies have noted that HCPs believe the most vital aspect of patient management for CKD is to improve the coordination and referral between primary and tertiary HCPs and use a multidisciplinary team approach to improve care quality [22,23,27,31]. However, in this study, only 7 HCP apps provided consultation management functions and only 1 contained a teleconsultation function. Therefore, current HCP apps are insufficient for supporting coordination and referral between HCPs.

The core value of patient-centered continual care is the continual provision of care that meets the needs of patients. In these terms, 38 apps provided daily patient records with informational continuity. Only 5 apps were able to deliver the information directly to HCPs to provide patients with management continuity. Moreover, 2 apps could only generate reports for delivery by email or other methods. Most of the apps were based on one-way patient education. Some apps had social media features, which can provide educational, social, and emotional support through interaction between HCPs and patients. This connectivity can be crucial for maintaining relationship continuity for people with chronic diseases. No app was found to provide any shared decision-making aid, the primary feature of patient-centered care, or to support engagement for multidisciplinary HCPs and primary caregivers. These findings reveal that current apps are not designed to support continuity of patient-centered care for CKD. The relational continuity of multidisciplinary HCPs is important in patient care for CKD. Patients have different expectations of continual care depending on the type and setting of care [3]. Before designing mobile apps, designers should consider the theoretical framework, clinical situation, and expectations of different users.

Apps on 360 Mobile Assistant were all free for download, and the most common languages were English and simplified Chinese. However, these apps were not suitable for traditional Chinese speakers. Since April 2016, many new and entirely different to former CKD-related apps have been released in China. All of these apps were developed by a hospital in collaboration with a medical device or app developer, were released with corresponding patient and HCP apps, required registration, included iOS and Android versions, and were simultaneously released on the App Store and 360 Mobile Assistant. This reveals China has attached increasing importance to the development of CKD-related apps. However, we found that some apps developed in China provided comprehensive CKD knowledge without describing the reference resources. Other researchers have also raised similar observations in mobile apps for cardiovascular disease in China [49,53]. Although some of these apps seem to provide information or advice regarding diagnosis, prognosis, monitoring, and treatment for HCPs and patients, none of the apps state in their introduction whether their function meets the definition of a medical device. We recommend that once the functionality and usage of an app fell into the scope of medical devices or in vitro diagnostic devices [46], it should be developed in accordance with the MEDDEV 2.1/6 guidelines and adhered to the requirements of EU Directive 93/42/EEC on medical devices [47]. Apps should have clear explanations regarding their medical device status in introductions on app platforms.

Larsen et al conducted a longitudinal study of mental health apps; they noted that 50% of apps change after 4 months and 1 app is removed every 2.9 days [44]. In this study’s follow-up analysis conducted in June 2017, the update rate of all apps was 11.8% (19/161) over 4 months. Moreover, 4 apps were removed from listings and 8 new apps were added. The frequency of change was much lower than that found by Larson et al for mental health apps. We also noted that 26 patient apps (23.64%) were single-function designs with high homogeneity, especially those apps that supported calculations such as eGFR and CCR. These apps usually refer to fixed formulas (eg, Cockcroft-Gault and Modification of Diet in Renal Disease) and simply alter the user interface. Because the functionality is simple and fixed, the frequency of updates is low. Apps with higher update frequency are mostly associated with CKD information for HCPs. Due to the frequency with which medical evidence is updated, updates occurred more often. In addition, apps related to self-management of CKD patients, such as CKD information, CKD nutrition education, and CKD self-management, were mostly developed by non-HCP organizations without statements of evidence-based references. This suggests that, although current apps related to self-management of CKD patients may have some self-managing functionalities, helping patient self-management without applying evidence-based medicine is prevalent. In the future, developers should apply evidence-based medicine when designing a patient-centered holistic solution for CKD.

### Limitations

This research was based on the public information provided by each platform. Therefore, we were unable to directly connect to users’ data to further understand their specific situations. We searched the 3 app platforms as thoroughly as possible to complete the baseline survey; however, due to rapid changes in the app platforms, we were unable to guarantee that no app was missed in the baseline survey. In addition, we only investigated and analyzed apps released on the App Store, Google Play, and 360 Mobile Assistant. We did not explore apps released on other channels, such as websites. We were also unable to determine how many apps released on other channels. Because comparing the quality of apps with different functionalities is complicated, we focused on exploring the functionalities of apps without judging the mobile app quality.

### Conclusions

In this study, we determined that the majority of patient-centered care mobile apps for CKD on popular app platforms contain calculation functions with one or more indicators. These popular app platforms do not provide sufficient information or medical evidence to help users choose apps. Currently, CKD-related mobile health apps have insufficient functionalities for both patient self-management and HCP management of patient care. A holistic solution is required that considers disease characteristics based on theoretical frameworks, clinical situations, and the needs of different users to enhance the continuity of patient-centered care. Furthermore, developers should follow uniform policies or regulatory standards from various internationally recognized organizations to ensure that apps meet the criteria of a medical device or in vitro diagnostic equipment; moreover, detailed descriptions should be provided for all apps.

## Appendix

Multimedia Appendix 1 Overview of mobile chronic kidney disease (CKD) patient-centered care related apps (N=177).

Multimedia Appendix 2 List of detailed features for each app.

## Abbreviations

BMI: creatinine clearance rate
BSA: body surface area
CCR: creatinine clearance rate
CKD: chronic kidney disease
eGFR: estimated glomerular filtration rate
FDA: US Food and Drug Administration
HCP: health care professional
IBW: ideal body weight
