# Supplementary material for: Mobile Health to Maintain Continuity of Patient-Centered Care for Chronic Kidney Disease: Content Analysis of Apps
Source: JMIR Mhealth Uhealth. 2018 Apr 20;6(4):e10173. doi: 10.2196/10173 (PMC5935804; doi:10.2196/10173)
Supplement: Multimedia Appendix 1 [file mhealth_v6i4e10173_app1.pdf]

Categories		All (N=177)	App Sore (N=103)	Google Pay (N=110)	360 Mobile Assistant (N=62)
Information provided by the developer					
Languages, n (%)					
	English	92 (52.0)	45 (43.7)	68 (61.8)	29 (47)
	Simple Chinese	33 (18.6)	25 (24.3)	1 (0.9)	21 (34)
	Multiple languages[a]	16 (9.0)	14 (13.6)	11 (10.0)	4 (6)
	Japanese	4 (2.3)	4 (3.9)	2 (1.8)	0 (0)
	Portuguese	6 (3.4)	4 (3.9)	5 (4.6)	1 (2)
	Spanish	7 (4.0)	3 (2.9)	7 (6.4)	1 (2)
	Traditional Chinese	7 (4.0)	3 (2.9)	6 (5.5)	4 (6)
	Korean	5 (2.8)	2 (1.9)	5 (4.6)	1 (2)
	French	3 (1.7)	1 (1.0)	2 (1.8)	1 (2)
	Russian	3 (1.7)	1 (1.0)	3 (2.7)	0 (0)
	German	1 (0.6)	1 (1.0)	0 (0.0)	0 (0)
Price (US $), n (%)					
	0	123 (69.5)	62 (60.2)	81 (73.6)	62 (100)
	1-100	22 (12.4)	17 (16.5)	10 (9.1)	0 (0)
	101-200	5 (2.8)	2 (1.9)	5 (4.6)	0 (0)
	>201	4 (2.3)	3 (2.9)	3 (2.7)	0 (0)
	Unknown	23 (13.0)	19 (18.5)	11 (10.0)	0 (0)
Registration requirements, n (%)					
	Yes	72 (40.7)	55 (53.4)	33 (30.0)	21 (34)
	No	105 (59.3)	48 (46.6)	77 (70.0)	41 (66)
Content rating[b], n (%)					
	High maturity	N/A[c]	0 (0)	38 (34.6)	N/A
	3+	N/A	0 (0)	72 (65.5)	N/A
	4+	N/A	47 (45.6)	0 (0.0)	N/A
	12+	N/A	22 (21.4)	0 (0.0)	N/A
	17+	N/A	34 (33.0)	0 (0.0)	N/A
Number of downloads, n (%)					
	0-100	N/A	N/A	26 (23.6)	43 (69)
	101-1000	N/A	N/A	29 (26.4)	14 (23)
	1001-10,000	N/A	N/A	41 (37.3)	5 (8)
	10,000-100,000	N/A	N/A	9 (8.2)	0 (0)
	>100,000	N/A	N/A	5 (4.6)	0 (0)
Privacy policy, n (%)					
	Yes	N/A	N/A	19 (17.3)	N/A
	No	N/A	N/A	91 (82.7)	N/A

**Categories**		**All (N=177)**	**App Sore (N=103)**	**Google Pay (N=110)**	**360 Mobile Assistant (N=62)**
Registration requirements, n (%)					
	Yes	72 (40.7)	55 (53.4)	33 (30.0)	21 (34)
	No	105 (59.3)	48 (46.6)	77 (70.0)	42 (66)
Description for certification of medical app[d], n (%)					
	Yes	0 (0.0)	0 (0.0)	0 (0.0)	0 (0)
	No	177 (100.0)	103 (100.0)	110 (100.0)	62 (100)
Users' rating					
Current rating (the average score), n (%)					
	≤3	N/A	N/A	4 (3.6)	N/A
	3.1-4	N/A	N/A	20 (18.2)	N/A
	4.1-5	N/A	N/A	32 (29.1)	N/A
	Unknown	N/A	N/A	54 (49.1)	N/A
Number of ratings, n (%)					
	0-100	N/A	N/A	102 (92.7)	N/A
	101-1000	N/A	N/A	5 (4.6)	N/A
	>1000	N/A	N/A	3 (2.7)	N/A
Researchers evaluation					
Recommended users, n (%)					
	HCPs[e]	67 (37.9)	47 (45.6)	48 (43.6)	18 (29)
	Patients	67 (37.9)	40 (38.8)	31 (28.2)	30 (48)
	Both	43 (24.3)	16 (15.5)	31 (28.2)	14 (23)
Description of reference, n (%)					
	Yes	142 (80.2)	79 (76.7)	100 (90.9)	51 (82)
	No	35 (19.8)	24 (23.3)	10 (9.1)	11 (18)
Developers, n (%)					
	Medical profession-related organizations	29 (16.4)	20 (19.4)	16 (14.6)	13 (11)
	Non-medical profession-related organizations	148 (83.6)	83 (80.6)	94 (85.5)	49 (79)
Paired app suite[f], n (%)		14 (12.7)	14 (13.6)	0 (0.0)	10 (16)

[a]The app was displayed in more than one language.

[b]Content ratings for apps can help users to understand an app's maturity.

[c]"N/A" means no suitable reference information on the app platform.

[d]There is a description of the medical app certification issued by any government agency.

[e]HCP: health care professionals.

[f]An app suite with patient and HCPs interoperability.
