# Supplementary material for: Mobile Health to Maintain Continuity of Patient-Centered Care for Chronic Kidney Disease: Content Analysis of Apps
Source: JMIR Mhealth Uhealth. 2018 Apr 20;6(4):e10173. doi: 10.2196/10173 (PMC5935804; doi:10.2196/10173)
Supplement: Multimedia Appendix 2 [file mhealth_v6i4e10173_app2.pdf]

Name and link (from first platform at the next column) of apps	Platforms			Functionalities of apps	Recommended users			Aspects of CKD patient self-management										Score of apps
	Google Paly	Apple Store	360 Mobile Assistant		Patients	HCPs	Both	Disease-specific knowledge	Managing medications	Engaging and sustaining social support	Maintaining social and occupational roles	Modifying lifestyle	Developing and sustaining a positive attitude and caring for mental and physical wellbeing	Building and sustaining effective relationships with healthcare providers	Establishing routine and planning ahead	Actively participating in healthcare	Recognising and effectively responding to symptoms	
H2O Overload	V	V	V	CKD self-management, CKD information, Various reminders, CKD nutrition education	V			V	V			V	V	V	V	V	V	8
ConQr	V		V	CKD self-management , Various reminders, CKD information	V			V	V				V	V	V	V	V	7
lose kidney fat app		V		CKD self-management, CKD nutrition education, CKD information	V			V	V			V	V		V	V	V	7
MiKidney	V	V		CKD self-management , Various reminders, CKD information	V			V	V			V	V		V	V	V	7
U肾		V	V	CKD self-management, E-consultation, CKD information, Social media	V			V		V			V	V	V	V	V	7
圣卫士病患端		V	V	CKD information, CKD nutrition education, CKD self-management, Social media, Medicine information, Activities news	V				V	V		V	V		V	V	V	7
江苏肾脏		V	V	E-consultation, CKD information, CKD self-management, Various reminders	V			V	V				V	V	V	V	V	7
腎好 iCKD	V	V	V	CKD self-management, CKD information, Medicine information, Various reminders, Medical resources inquiries	V			V	V				V	V	V	V	V	7
腎利人生	V			CKD self-management, Medical resources inquiries, Activities news, CKD information	V			V		V			V	V	V	V	V	7
MyKidneyCare	V		V	CKD self-management, E-appointment, E-consultation, CKD information	V			V					V	V	V	V	V	6
Парацельс (Контроль давления)	V			CKD self-management, eGFR calculation	V			V	V			V	V		V	V		6
心云肾健康		V		CKD self-management, CKD information	V			V	V				V		V	V	V	6
优医-患者		V		CKD self-management , CKD information, E-consultation	V			V					V	V	V	V	V	6
掌上肾医		V		CKD self-management, E-consultation, CKD information	V			V					V	V	V	V	V	6
腎好 iCKD Lite	V	V	V	CKD self-management, CKD information, Various reminders, Medicine information	V			V	V				V		V	V	V	6
爱肾		V	V	eGFR calculation, CKD nutrition education, Social media, E-consultation	V			V		V		V	V	V		V		6
肾病咨询中心患者端		V	V	CKD self-management, CKD information, E-consultation	V			V					V	V	V	V	V	6
Phosphorus Tracker	V	V		CKD nutrition education, CKD self-management	V			V				V	V		V	V		5

Name and link (from first platform at the next column) of apps	Platforms			Functionalities of apps	Recommended users			Aspects of CKD patient self-management										Score of apps
	Google Paly	Apple Store	360 Mobile Assistant		Patients	HCPs	Both	Disease-specific knowledge	Managing medications	Engaging and sustaining social support	Maintaining social and occupational roles	Modifying lifestyle	Developing and sustaining a positive attitude and caring for mental and physical wellbeing	Building and sustaining effective relationships with healthcare providers	Establishing routine and planning ahead	Actively participating in healthcare	Recognising and effectively responding to symptoms	
RENAL TRKRR	V		V	CKD self-management	V				V			V	V		V	V		5
RenalHelp	V			CKD self-management, Medicine information	V				V			V	V		V	V		5
Smart GFR Tracker	V			eGFR calculation, CKD staging, CKD information, CKD self-management	V			V					V		V	V	V	5
小K管家-肾病检查记录/康复日志/知识分享	V	V	V	CKD self-management, CKD information, eGFR calculation	V			V					V		V	V	V	5
만성콩팥병 관리수첩	V	V		CKD self-management, eGFR calculation, CKD information, CKD knowledge test	V			V					V		V	V	V	5
인 섭취 다이어리	V	V		Nutrition calculation, CKD nutrition education, CKD self-management, Various reminders	V			V				V	V		V	V		5
콩팥병 식이	V			CKD self-management, CKD nutrition education	V			V				V	V		V	V		5
肾斗士	V	V	V	CKD self-management, eGFR calculation, CKD information, Privacy management	V			V					V		V	V	V	5
肾博士			V	CKD self-management , Social media, E-consultation, Emergency call	V					V			V	V	V	V		5
Calculadora GFR CKD-EPI	V	V		eGFR calculation, eGFR information, Report generator			V	V						V		V	V	4
Caution Calcultor		V		CKD self-management	V							V	V		V	V		4
CRN Pocket Guide		V		CKD self-management	V							V	V		V	V		4
Dieta renal-1	V		V	CKD nutrition education, eGFR calculation, CKD staging, CKD information	V			V				V				V	V	4
Jinzou		V		CKD self-management, eGFR calculation	V							V	V		V	V		4
kidneyLuv	V			CKD self-management	V							V	V		V	V		4
My Food Coach	V	V	V	CKD nutrition education, CKD self-management	V							V	V		V	V		4
nephrotic syndrome	V	V		CKD self-management, Report generator	V								V	V	V	V		4
Pocket Dietitian	V			CKD nutrition education, CKD self-management	V							V	V		V	V		4

Name and link (from first platform at the next column) of apps	Platforms			Functionalities of apps	Recommended users			Aspects of CKD patient self-management										Score of apps
	Google Paly	Apple Store	360 Mobile Assistant		Patients	HCPs	Both	Disease-specific knowledge	Managing medications	Engaging and sustaining social support	Maintaining social and occupational roles	Modifying lifestyle	Developing and sustaining a positive attitude and caring for mental and physical wellbeing	Building and sustaining effective relationships with healthcare providers	Establishing routine and planning ahead	Actively participating in healthcare	Recognising and effectively responding to symptoms	
Rapid GFR	V			CCR calculation, eGFR calculation, CKD staging, CKD self-management	V								V		V	V	V	4
Renal Dose	V		V	eGFR calculation, CKD self-management	V				V				V		V	V		4
我的健康秘書	V		V	CKD self-management, CKD staging	V								V		V	V	V	4
京东誉美肾病医院			V	E-appointment, E-consultation, CKD information	V			V						V		V	V	4
补肾宝典		V		CKD information, E-consultation	V			V						V		V	V	4
肾病		V		CKD information, E-consultation	V			V						V		V	V	4
肾病			V	E-appointment, E-consultation, CKD information	V			V						V		V	V	4
肾病好医生			V	E-appointment, E-consultation, CKD information	V			V						V		V	V	4
肾病治疗			V	E-appointment, E-consultation, CKD information	V			V						V		V	V	4
肾病问医生-在线咨询			V	E-appointment, E-consultation, CKD information	V			V						V		V	V	4
肾康		V		E-appointment, E-consultation, CKD information	V			V						V		V	V	4
肾臓病用かんたん食事管理アプリ　腎録		V		CKD self-management	V							V	V		V	V		4
CKD數據管理		V		CKD self-management	V								V		V	V		3
My Kidneys, My Health handbook	V	V		CKD information, CKD nutrition education	V			V				V					V	3
Socialvida	V	V	V	eGFR calculation, Social media	V					V			V			V		3
Zero & Low Sodium Foods	V			CKD nutrition education, Nutrition calculation, Body Mass Index (BMI) calculation			V	V				V				V		3
小児CKD-eGFR计算	V	V		eGFR calculation, eGFR information			V	V								V	V	3
腎病指標檢測	V		V	eGFR calculation, CKD staging, CKD information	V			V								V	V	3

Name and link (from first platform at the next column) of apps	Platforms			Functionalities of apps	Recommended users			Aspects of CKD patient self-management										Score of apps
	Google Paly	Apple Store	360 Mobile Assistant		Patients	HCPs	Both	Disease-specific knowledge	Managing medications	Engaging and sustaining social support	Maintaining social and occupational roles	Modifying lifestyle	Developing and sustaining a positive attitude and caring for mental and physical wellbeing	Building and sustaining effective relationships with healthcare providers	Establishing routine and planning ahead	Actively participating in healthcare	Recognising and effectively responding to symptoms	
Calculadora De Función Renal	V			eGFR calculation, CKD staging, Ideal Body Weight (IBW) calculation, Body Surface area (BSA) calculation			V									V	V	2
CKD Risk Calc Free	V	V	V	eGFR calculation, Body Surface area (BSA) calculation, Ideal Body Weight (IBW) calculation, CKD staging			V									V	V	2
Clinical Lab (Gault GFR)	V		V	eGFR calculation, CKD staging			V									V	V	2
Cockcroft-Gault CrCl	V		V	CCR calculation, CCR severity grading			V									V	V	2
eGFR Calculator	V		V	eGFR calculation, CKD staging, eGFR information			V	V									V	2
eGFR Calculator	V		V	eGFR calculation, CKD staging			V									V	V	2
eGFR Calculators	V	V	V	eGFR calculation, eGFR information, CKD information			V	V									V	2
Função Renal	V	V		eGFR calculation, CKD staging			V									V	V	2
Função Renal - RenalCALC	V			eGFR calculation, CKD staging			V									V	V	2
GFR & BSA Calculator	V			eGFR calculation, CKD staging			V									V	V	2
Healthy Kidneys Grocery List		V		CKD nutrition education	V			V				V						2
Kidney diet Foods		V		Nutrition calculation, CKD nutrition education	V			V				V						2
Kidney diet Recipes		V		CKD nutrition education	V			V				V						2
Kidney Disease & Symptoms	V			CKD information	V			V									V	2
Kidney Disease Support : Tips and Daily Help		V		CKD information	V			V									V	2
Kidney Options		V		CKD information	V			V									V	2
Multiple Creatinine Clearance	V			eGFR calculation, Ideal Body Weight (IBW) calculation, Body Mass Index (BMI) calculation			V	V									V	2
NewtriCalc	V	V		eGFR calculation, Nutrition calculation, Body Mass Index (BMI) calculation, Body Surface area (BSA) calculation, Ideal Body Weight (IBW) calculation			V					V				V		2

Name and link (from first platform at the next column) of apps	Platforms			Functionalities of apps	Recommended users			Aspects of CKD patient self-management										Score of apps
	Google Paly	Apple Store	360 Mobile Assistant		Patients	HCPs	Both	Disease-specific knowledge	Managing medications	Engaging and sustaining social support	Maintaining social and occupational roles	Modifying lifestyle	Developing and sustaining a positive attitude and caring for mental and physical wellbeing	Building and sustaining effective relationships with healthcare providers	Establishing routine and planning ahead	Actively participating in healthcare	Recognising and effectively responding to symptoms	
Rapid GFR Free	V		V	CCR calculation, eGFR calculation, CKD staging			V									V	V	2
RenalTouchXL		V		CKD information	V			V									V	2
Texa de Filtracao Glomerular		V		eGFR calculation, CKD staging			V									V	V	2
中国肾病网		V		CKD information	V			V									V	2
对比剂肾病风险评估软件			V	eGFR calculation, Risk assessment	V											V	V	2
爱肾宝典	V		V	CKD information	V			V									V	2
肾不好的症状			V	CKD information	V			V									V	2
肾好吧		V		Social media	V					V			V					2
肾病医生		V		E-appointment, E-consultation	V									V		V		2
肾病食疗菜谱		V	V	CKD nutrition education	V			V				V						2
肾脏护理技巧	V			CKD information	V			V									V	2
肾脏病的饮食揭密			V	CKD nutrition education	V			V				V						2
A1 Renal Calculator		V		CCR calculation			V									V		1
ANHAES eGFR calculator	V			eGFR calculation			V									V		1
Avaliação Renal	V			eGFR calculation			V									V		1
CalcCrcl		v		CCR calculation			V									V		1
CKD Calculator		V		eGFR calculation			V									V		1
CrCl	V			CCR calculation			V									V		1

Name and link (from first platform at the next column) of apps	Platforms			Functionalities of apps	Recommended users			Aspects of CKD patient self-management										Score of apps
	Google Paly	Apple Store	360 Mobile Assistant		Patients	HCPs	Both	Disease-specific knowledge	Managing medications	Engaging and sustaining social support	Maintaining social and occupational roles	Modifying lifestyle	Developing and sustaining a positive attitude and caring for mental and physical wellbeing	Building and sustaining effective relationships with healthcare providers	Establishing routine and planning ahead	Actively participating in healthcare	Recognising and effectively responding to symptoms	
Creatinine Clearance Cal	V			CCR calculation			V									V		1
eGFR Calc		V		eGFR calculation			V									V		1
eGFR Calc	V			eGFR calculation			V									V		1
eGFR Calculator	V			eGFR calculation			V									V		1
eGFR健康評估	V			eGFR calculation			V									V		1
gfr		V		eGFR calculation			V									V		1
GFR Calculator	V		V	eGFR calculation			V									V		1
glomerula filtration rate calculator		V		eGFR calculation			V									V		1
Iclearance		V		CCR calculation			V									V		1
MI TFG	V			eGFR calculation			V									V		1
NefroCalc		V		eGFR calculation			V									V		1
Nephro Cal Demo	V		V	eGFR calculation			V									V		1
Nephrology Clinical Tool	V			CCR calculation			V									V		1
Renal Function Calculator			V	eGFR calculation			V									V		1
Renal function calculator(肾功能计算器)	V		V	eGFR calculation			V									V		1
Renal function Free	V		V	CCR calculation			V									V		1
Калькулятор СКФ	V			eGFR calculation			V									V		1
肌酐清除率			V	CCR calculation			V									V		1

Name and link (from first platform at the next column) of apps	Platforms			Functionalities of apps	Recommended users			Aspects of CKD patient self-management										Score of apps
	Google Paly	Apple Store	360 Mobile Assistant		Patients	HCPs	Both	Disease-specific knowledge	Managing medications	Engaging and sustaining social support	Maintaining social and occupational roles	Modifying lifestyle	Developing and sustaining a positive attitude and caring for mental and physical wellbeing	Building and sustaining effective relationships with healthcare providers	Establishing routine and planning ahead	Actively participating in healthcare	Recognising and effectively responding to symptoms	
薬剂师の临床検査值计算器	V	V		CCR calculation, Body Surface area (BSA) calculation, eGFR calculation			V									V		1
肾小球滤过率			V	eGFR calculation			V									V		1
BS3 Nephrology Pack	V	V	V	Comprehensive clinical calculation (including eGFR), Clinical decision support for CKD		V												
Calculate by QxMD	V	V	V	Comprehensive clinical calculation (including eGFR)		V												
CalcuLithiase		V		eGFR calculation, Clinical decision support for CKD		V												
Chula Kidney Calculator	V		V	Comprehensive clinical calculation (including eGFR)		V												
CKD Go!	V	V		CKD staging, Clinical decision support for CKD, Other clinical decision support, CKD information		V												
CKD Risk Calc PRO	V	V		eGFR calculation, Body Surface area (BSA) calculation, Ideal Body Weight (IBW) calculation, CKD staging, eGFR information, Clinical decision support for CKD		V												
CKD Risk Map	V	V	V	Risk assessment, Clinical decision support for CKD		V												
Diabetes Clinical Care		V		Provision of CKD medical professional information		V												
Diabetic Nephropathy Management Support-DNMS		V		eGFR calculation, CKD staging, Clinical decision support for CKD		V												
eGFR	V	V		Comprehensive clinical calculation (including eGFR)		V												
eGFR Calculators Pro	V			eGFR calculation, CKD staging, Clinical decision support for CKD		V												
ESPN	V			Provision of CKD medical professional information		V												
Global Nephro Calculator		V		Comprehensive clinical calculation (including eGFR), CKD staging, Risk assessment		V												
Guías Clínicas ECNT INS MINSAL	V			Provision of CKD medical professional information		V												
IM Formulae	V			Comprehensive clinical calculation (including eGFR)		V												
KDIGO Mobile	V	V		Provision of CKD medical professional information		V												

Name and link (from first platform at the next column) of apps	Platforms			Functionalities of apps	Recommended users			Aspects of CKD patient self-management										Score of apps
	Google Paly	Apple Store	360 Mobile Assistant		Patients	HCPs	Both	Disease-specific knowledge	Managing medications	Engaging and sustaining social support	Maintaining social and occupational roles	Modifying lifestyle	Developing and sustaining a positive attitude and caring for mental and physical wellbeing	Building and sustaining effective relationships with healthcare providers	Establishing routine and planning ahead	Actively participating in healthcare	Recognising and effectively responding to symptoms	
Kidney News	V	V		Provision of CKD medical professional information		V												
KidneyWise Clinical Toolkit	V	V		eGFR calculation, Clinical decision support for CKD		V												
MedCalc 3000 Kidney	V	V		Comprehensive clinical calculation (including eGFR)		V												
Medfixation Medical Calculator	V	V		Comprehensive clinical calculation (including eGFR)		V												
Medical Calculators	V	V	V	Comprehensive clinical calculation (including eGFR)		V												
Medical Tools	V		V	Comprehensive clinical calculation (including eGFR)		V												
MediCalc Medical Calculator	V	V		Comprehensive clinical calculation (including eGFR)		V												
MediCalc®	V	V		Comprehensive clinical calculation (including eGFR)		V												
medsys GFR		V		eGFR calculation, Body Mass Index (BMI) calculation, Body Surface area (BSA) calculation		V												
MyCera	V		V	Clinical decision support for CKD		V												
NefroConsultor	V	V		eGFR calculation, CKD staging, Clinical decision support for CKD		V												
Nephro Cal	V			Body Surface area (BSA) calculation, Comprehensive clinical calculation (including eGFR)		V												
Nephro&Urology		V		CKD information		V												
NephroCalc™	V	V		Comprehensive clinical calculation (including eGFR)		V												
Nephrology Assistant Free	V		V	Comprehensive clinical calculation (including eGFR)		V												
nephrology calculator	V		V	Comprehensive clinical calculation (including eGFR)		V												
Nephrology On-Demand Plus	V	V		Provision of CKD medical professional information		V												
Nephrology pocket	V	V		eGFR calculation, Provision of CKD medical professional information		V												

Name and link (from first platform at the next column) of apps	Platforms			Functionalities of apps	Recommended users			Aspects of CKD patient self-management										Score of apps
	Google Paly	Apple Store	360 Mobile Assistant		Patients	HCPs	Both	Disease-specific knowledge	Managing medications	Engaging and sustaining social support	Maintaining social and occupational roles	Modifying lifestyle	Developing and sustaining a positive attitude and caring for mental and physical wellbeing	Building and sustaining effective relationships with healthcare providers	Establishing routine and planning ahead	Actively participating in healthcare	Recognising and effectively responding to symptoms	
Nephrology Tool by Epocrates		V		Comprehensive clinical calculation (including eGFR)		V												
NurseCalc - Nursing Calculator	V	V		Comprehensive clinical calculation (including eGFR)		V												
NutriGuides Mobile	V	V		Provision of CKD nutritional care professional information		V												
powerOne Medical Calculator	V			Comprehensive clinical calculation (including eGFR)		V												
PSCV	V	V		eGFR calculation, Clinical decision support for CKD		V												
Renal & Urology News	V	V		Provision of CKD medical professional information		V												
renal calc		V		eGFR calculation		V												
renal eval		V		eGFR calculation		V												
Roots of Life	V			Comprehensive clinical calculation (including eGFR)		V												
SAN	V	V		Provision of CKD medical professional information, eGFR calculation, Social media		V												
Score Index for Haemodialysis	V			CKD evaluation, Clinical decision support for CKD		V												
Screening for Albuminuria	V	V	V	CKD information, Other clinical decision support		V												
Seminars in Nephrology	V	V		Provision of CKD medical professional information		V												
SMARTfiches Néphrologie	V			Provision of CKD medical professional information, Comprehensive clinical calculation (including eGFR)		V												
SMARTfiches Néphrologie Free	V	V		Provision of CKD medical professional information, Comprehensive clinical calculation (including eGFR)		V												
Tamizaje - Nefrología	V			Risk assessment, Body Mass Index (BMI) calculation, eGFR calculation		V												
Tx Konnect	V		V	Provision of CKD medical professional information, eGFR calculation, CCR calculation		V												
UCI RenalCalc	V	V		Comprehensive clinical calculation (including eGFR)		V												

Name and link (from first platform at the next column) of apps	Platforms			Functionalities of apps	Recommended users			Aspects of CKD patient self-management										Score of apps
	Google Paly	Apple Store	360 Mobile Assistant		Patients	HCPs	Both	Disease-specific knowledge	Managing medications	Engaging and sustaining social support	Maintaining social and occupational roles	Modifying lifestyle	Developing and sustaining a positive attitude and caring for mental and physical wellbeing	Building and sustaining effective relationships with healthcare providers	Establishing routine and planning ahead	Actively participating in healthcare	Recognising and effectively responding to symptoms	
U肾医护端		V	V	Consultation management, Appointment reminder, Provision of CKD medical professional information		V												
WE APP	V			Comprehensive clinical calculation (including eGFR)		V												
КардиоЭксперт II	V			Comprehensive clinical calculation (including eGFR)		V												
Помощь врачу	V	V		Comprehensive clinical calculation (including eGFR)		V												
方剂	V		V	Comprehensive clinical calculation (including eGFR)		V												
圣卫士医生端		V	V	Consultation management, Patient management		V												
江苏肾脏医护端		V	V	Consultation management, Teleconsultation, Appointment management, Patient management		V												
掌上肾医医生端		V		Comprehensive clinical calculation (including eGFR), Clinical decision support for CKD		V												
신장학계산기2	V			Comprehensive clinical calculation (including eGFR)		V												
爱肾医护端		V	V	Consultation management, Appointment reminder, Provision of CKD medical professional information		V												
肾内科专家		V	V	Provision of CKD medical professional information, Clinical decision support for CKD		V												
肾病管理专家医师端		V	V	Patient management, Consultation management		V												
肾健康医生版		V		Patient management, Consultation management		V												
肾博士医生版		V		Patient management, Consultation management		V												
诊疗计算App for iPhone		V		Comprehensive clinical calculation (including eGFR)		V												
